# Paramagnetic rims are a promising diagnostic imaging biomarker in multiple sclerosis

**DOI:** 10.1177/13524585221118677

**Published:** 2022-08-26

**Authors:** Isobel Meaton, Amjad Altokhis, Christopher Martin Allen, Margareta A Clarke, Tim Sinnecker, Dominik Meier, Christian Enzinger, Massimiliano Calabrese, Nicola De Stefano, Alain Pitiot, Antonio Giorgio, Menno M Schoonheim, Friedemann Paul, Mikolaj A Pawlak, Reinhold Schmidt, Cristina Granziera, Ludwig Kappos, Xavier Montalban, Àlex Rovira, Jens Wuerfel, Nikos Evangelou

**Affiliations:** Mental Health and Clinical Neurosciences Academic Unit, School of Medicine, University of Nottingham, Nottingham, UK; Mental Health and Clinical Neurosciences Academic Unit, School of Medicine, University of Nottingham, Nottingham, UK; Mental Health and Clinical Neurosciences Academic Unit, School of Medicine, University of Nottingham, Nottingham, UK; Institute of Imaging Science, Vanderbilt University Medical Center, Vanderbilt University, Nashville, TN, USA; Medical Image Analysis Center AG and Department of Biomedical Engineering, University Basel, Basel, Switzerland; Medical Image Analysis Center AG and Department of Biomedical Engineering, University Basel, Basel, Switzerland; Department of Neurology, Medical University of Graz, Graz, Austria; Neurology Unit, Department of Neuroscience, Biomedicine and Movement Sciences, University of Verona, Verona, Italy; Department of Medicine, Surgery and Neuroscience, University of Siena, Siena, Italy; Laboratory of Image and Data Analysis, Ilixa Ltd, London, UK; Department of Medicine, Surgery and Neuroscience, University of Siena, Siena, Italy; Department of Anatomy and Neurosciences, Amsterdam Neuroscience, Amsterdam UMC, Vrije Universiteit Amsterdam, Amsterdam, the Netherlands; Neurocure Clinical Research Center, Charité-Universitätsmedizin Berlin, Corporate Member of Freie Universität Berlin, Humboldt-Universität zu Berlin and Berlin Institute of Health, Berlin, Germany; Department of Neurology and Cerebrovascular Disorders, Poznan University of Medical Sciences, Poznan, Poland; Department of Neurology, Medical University of Graz, Graz, Austria; Research Center for Clinical Neuroimmunology and Neuroscience Basel (RC2NB), Departments of Head, Spine and Neuromedicine, Clinical Research and Biomedical Engineering, University Hospital, University of Basel, Basel, Switzerland; Research Center for Clinical Neuroimmunology and Neuroscience Basel (RC2NB), Departments of Head, Spine and Neuromedicine, Clinical Research and Biomedical Engineering, University Hospital, University of Basel, Basel, Switzerland; Centre d’Esclerosi Multiple de Catalunya (Cemcat), Department of Neurology/Neuroimmunology, Hospital Universitari Vall d’Hebron, Universitat Autonoma de Barcelona, Barcelona, Spain; Section of Neuroradiology, Department of Radiology, Hospital Universitari Vall d’Hebron, Universitat Autònoma de Barcelona, Barcelona, Spain; Medical Image Analysis Center AG and Department of Biomedical Engineering, University Basel, Basel, Switzerland/Neurocure Clinical Research Center, Charité-Universitätsmedizin Berlin, Corporate Member of Freie Universität Berlin, Humboldt-Universität zu Berlin and Berlin Institute of Health, Berlin, Germany; Mental Health and Clinical Neurosciences Academic Unit, School of Medicine, University of Nottingham, Nottingham, UK

**Keywords:** Multiple sclerosis, MRI, CIS, biomarkers

## Abstract

**Background:**

White matter lesions (WMLs) on brain magnetic resonance imaging (MRI) in multiple sclerosis (MS) may contribute to misdiagnosis. In chronic active lesions, peripheral iron-laden macrophages appear as paramagnetic rim lesions (PRLs).

**Objective:**

To evaluate the sensitivity and specificity of PRLs in differentiating MS from mimics using clinical 3T MRI scanners.

**Method:**

This retrospective international study reviewed MRI scans of patients with MS (*n* = 254), MS mimics (*n* = 91) and older healthy controls (*n* = 217). WMLs, detected using fluid-attenuated inversion recovery MRI, were analysed with phase-sensitive imaging. Sensitivity and specificity were assessed for PRLs.

**Results:**

At least one PRL was found in 22.9% of MS and 26.1% of clinically isolated syndrome (CIS) patients. Only one PRL was found elsewhere. The identification of ⩾1 PRL was the optimal cut-off and had high specificity (99.7%, confidence interval (CI) = 98.20%–99.99%) when distinguishing MS and CIS from mimics and healthy controls, but lower sensitivity (24.0%, CI = 18.9%–36.6%). All patients with a PRL showing a central vein sign (CVS) in the same lesion (*n* = 54) had MS or CIS, giving a specificity of 100% (CI = 98.8%–100.0%) but equally low sensitivity (21.3%, CI = 16.4%–26.81%)

**Conclusion:**

PRLs may reduce diagnostic uncertainty in MS by being a highly specific imaging diagnostic biomarker, especially when used in conjunction with the CVS.

## Introduction

The need for accurate, early diagnosis and consideration of early treatment of multiple sclerosis (MS) introduces challenges for clinicians.^1^ The 2017 modified McDonald diagnostic criteria^2^ necessitate typical clinical symptoms and the presence of white matter lesions (WMLs) on magnetic resonance imaging (MRI). These criteria shorten time to diagnosis^3^ and improve the sensitivity of diagnosing MS.^4^ Yet misdiagnosis is still common,^5,6^ especially when the MRI criteria are incorrectly applied outside of a typical clinical presentation or when there is incorrect interpretation of MRI findings. It has been suggested that recent changes to the MRI criteria decreased the diagnostic specificity,^7–9^ as WMLs can be present in other conditions such as migraine,^10^ neuromyelitis optica spectrum disorder (NMOSD)^11^ and central nervous system vasculitis.^12^

There is growing acceptance of the role of the central vein sign (CVS) in diagnosing MS^13^ leading to increased use of phase-sensitive imaging at the time of first clinical presentation.^14,15^

Some chronic MS lesions have persistent active demyelination, the products of which are engulfed within activated microglia/macrophages on the periphery of the lesion. One such product is ferrous iron released into the extracellular space during the destruction of oligodendrocytes.^16,17^ This can be detected in vivo with phase-sensitive imaging where it presents as a paramagnetic rim (PR).^16,17^ Paramagnetic rim lesions (PRLs) appear as a hypointense, ring-like structures that surround WML on phase-sensitive MRI sequences. PRLs may increase in size whereas non-PRLs decrease in size or remain unchanged.^18,19^

This imaging marker has been studied in detail using 7 Tesla (T) MRI.^19–22^ Importantly, 3T MRI studies have also detected PRLs in MS^23–25^ and corroborated the possible diagnostic and prognostic value.^26,27^ However, there are reservations on the clinical utility as PRLs are only seen in a minority of WMLs.^13,28^

This retrospective international, multicentre study within the Magnetic Resonance Imaging in MS (MAGNIMS) Study Group aimed to test the potential for PRLs in clinical practice. MRIs for patients with MS and MS mimics (including cerebral small vessel disease, migraine and NMOSD) were compared. This dataset was originally collected by Sinnecker et al.^14^ to evaluate the value of CVS in MS.

## Methods

### Participants

The study included 562 participants scanned at 7 MS centres across Europe between 2010 and 2016. The participants were enrolled in ongoing observational studies or included in neuroimaging research databases, all of which were approved by the institutional review board at each centre. All patients provided written informed consent prior to MRI. The inclusion criteria, diagnostic criteria and patient demographics have been reported previously.^16^ All patients with NMOSD had antibodies against aquaporin 4.^14^ Susceptibility-weighted imaging (SWI) and three-dimensional (3D) fluid-attenuated inversion recovery (FLAIR) scans acquired at 3T of sufficient quality were analysed. Scan acquisition details for each centre can be found in the supplementary materials of Sinneker et al.^16^

### Image post-processing

FLAIR images from each participant were co-registered to the SWI using the ITK registration library (Insight Software Consortium), which was implemented in 3D Slicer, version 4.6.2 (Slicer Community). Insufficient co-registration resulted in exclusion from analysis. The registered images were then sectioned into eight equal-sized 3D blocks to ensure blinding of assessors to the patients’ diagnosis.^14^

### Image analysis

All image analysis was performed by two trained investigators (A.A. and I.M.) using 3D Slicer (version 4.11.2). Each 3D block was reviewed by A.A. or I.M. and results were collated after all image analysis was performed to avoid lesion classification in one part of a brain influencing assessment of other regions of the same brain. The supratentorial regions of the FLAIR MRI scans were analysed for WMLs with a long axis ⩾ 3 mm. Lesions were classified based on their location as cortical/juxtacortical (in direct contact with the cerebral cortex), periventricular (in direct contact with the lateral/third ventricles), deep WML (not in direct contact with the cortex or ventricles) or in direct contact with deep grey matter structures.^29^

**Figure 1.**
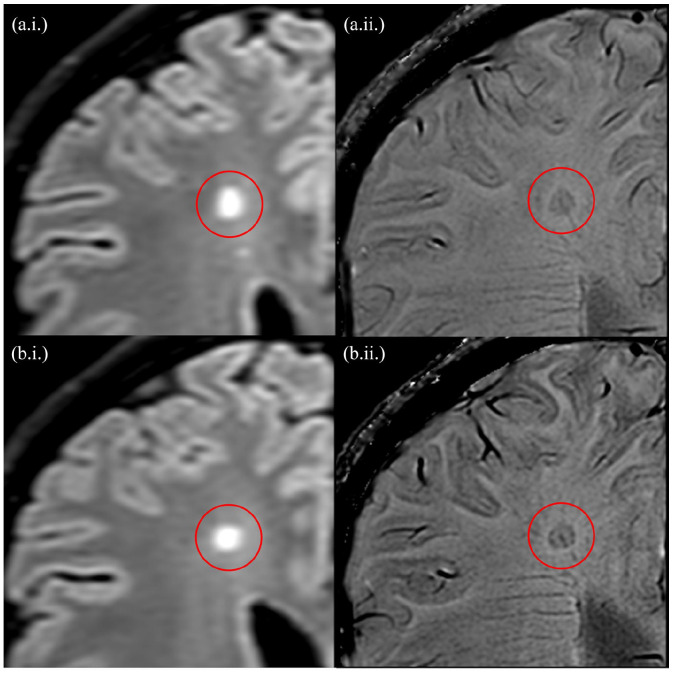
Consecutive slices of a paramagnetic rim lesion (with a central vein) detected using the fluid-attenuated inversion recovery (a.i. and b.i.) and phase-sensitive imaging (a.ii. and b.ii.), at 3T. As per the study protocol, the lesions demonstrate a hypointense, ring-like structure corresponding to the lesion edge which is present on at least two consecutive slices.

The SWI scans were then analysed for the presence of PRLs. A PRL was defined as a hypointense, ring-like structure on phase-sensitive imaging. The rim had to correspond to the WML edge on the FLAIR scan, encircle it fully or partially and must be visible on at least two consecutive image slices (Figure 1). As part of this study, CVS was also analysed using the North American Imaging in MS Cooperative (NAIMS) criteria.^30^

### Quality assessment

Each block was assessed for artefacts and co-registration quality of FLAIR and SWI before the detection of WMLs. A total of 18 out of 5196 blocks failed this quality test and were excluded from the analysis. Once image analysis was completed, the blocks were de-anonymised and matched to patient data.

### Statistical analysis

The statistical analysis was performed using IBM SPSS statistics, version 20 (IBM). Sensitivity and specificity were calculated for having at least one PRL per complete scan and presented with 95% confidence intervals (CIs). A secondary analysis was performed, only considering lesions that demonstrated both the PRL and CVS; this was also presented as sensitivity and specificity with 95% CI. Then, a sequential analysis was performed that first checks for the PRL and then, if no PRL is detected, checks for CVS across the entire scan. A chi-square test was performed to investigate the location of PRL and WMLs (deep white matter vs all other locations).

The logistic regression was used to produce receiver-operating characteristic (ROC) curves. In the logistic model, diagnosis (MS vs MS-mimics) was set as a dependent variable and PRL (or CVS) as an independent variable. The ROC curve is a plot of sensitivity against 1 − specificity. The sensitivity and specificity values were obtained by varying the cut-off to dichotomise PRL (or CVS) (S1).

### Interrater reliability

Interrater and intra-rater reliability for lesion identification and PRL detection was assessed in a randomly selected enriched data set of 100 blocks (53 with a PRL and 47 without) containing MS and non-MS lesions. Reliability was calculated using Cohen’s Kappa.

## Results

### Cohort description

The demographics of the 562 participants (182 males and 380 females) are shown in Table 1.

**Table 1.** Overview of participants and analysed lesions.

	MS	CIS	Cluster headache	Migraine	SLE	NMOSD	Diabetes mellitus	Ageing healthy controls	Total
Number of patients (female)	166 (110)	88 (59)	3 (1)	21 (18)	19 (16)	30 (26)	18 (10)	217 (140)	562 (380)
Mean age [SD]	37.3 [7.4]	32.6 [7.7]	49.3 [12.6]	40.8 [8.7]	32.5 [9.5]	46.5 [11.8]	68.3 [14.0]	64.1 [18.1]	48.4 [17.8]
Symptom duration, mean (range)	6.5 (0–30.5)	0.3 (0–2.5)	–	–	–	2.9 (0–9.6)	–	–	–
White matter lesions analysed									
No. of patients with ⩾1 WML, (%)	164 (98.8)	84 (95.4)	2 (66.7)	21 (100.0)	15 (78.9)	18 (60.0)	18 (100)	163 (75.1)	485 (86.3)
No. of lesions	3065	922	19	266	77	171	355	1142	6017
Median no. per patient (range)	17 (0–61)	7 (0–54)	9.5 (4–15)	10 (1–38)	4 (0–12)	1 (0–38)	17.5 (1–66)	3 (0–30)	7 (0–66)
Paramagnetic rim lesions									
No. of patients with ⩾1 PRL, (%)	38 (22.9)	23 (26.1)	0	0	0	0	1 (5.6)	0	62 (11.3)
No. of lesions (%)	72 (2.3)	57 (6.2)	0	0	0	0	1 (0.3)	0	130 (2.2)
Median no. per patient (range)	0 (0–6)	0 (0–8)	0	0	0	0	0 (0–1)	0	0 (0–8)

MS: multiple sclerosis; CIS: clinically isolated syndrome; NMOSD: neuromyelitis optica disorder; SD: standard deviation; SLE: systematic lupus erythematosus; WML: white matter lesion; PRL: paramagnetic rim lesions.

### Lesion count and distribution

**Figure 2.**
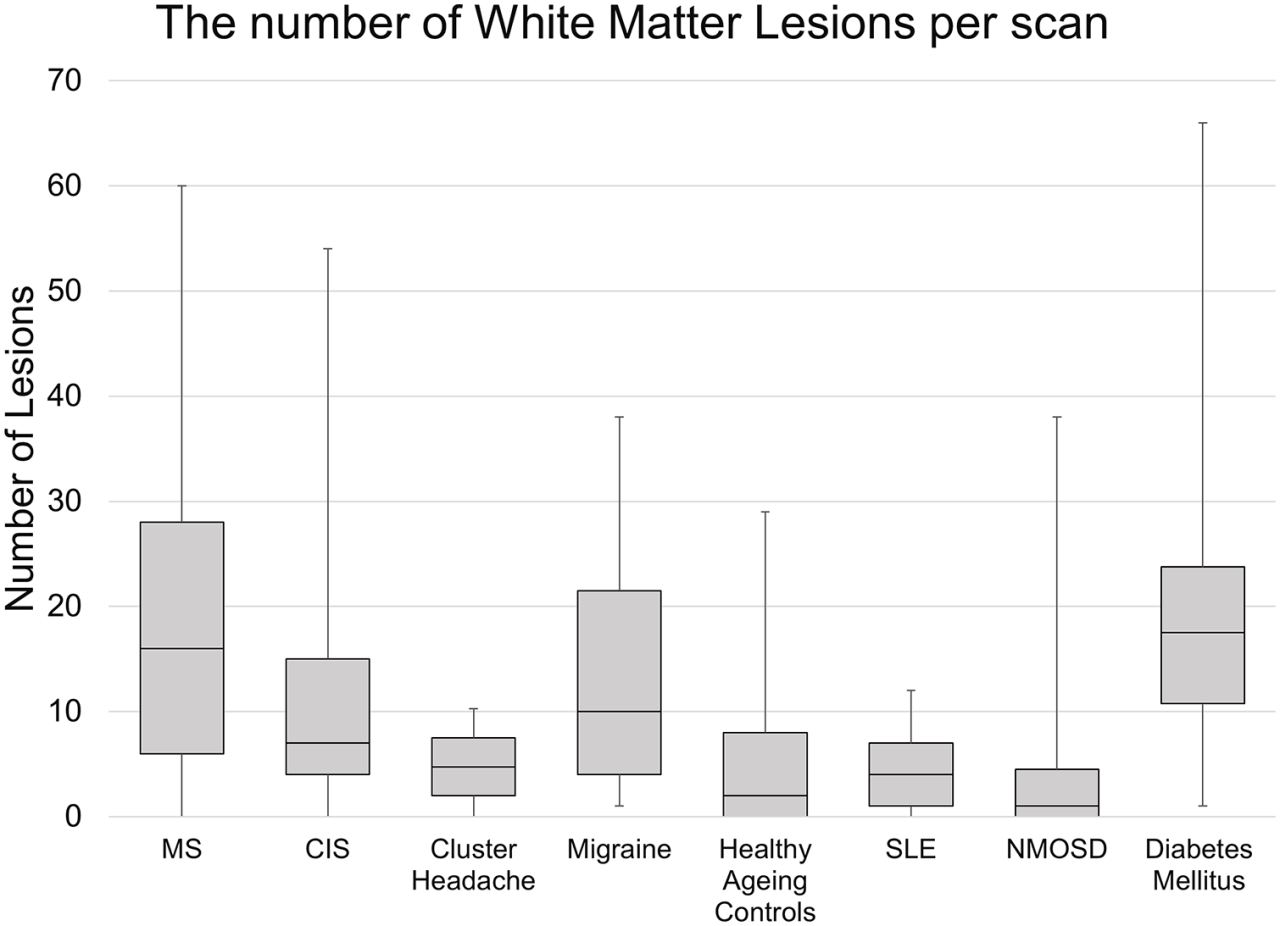
A box and whisker plot showing the mean and interquartile ranges of the number of white matter lesions per patient in each condition analysed. NMOSD: neuromyelitis optica spectrum disorder; Control: healthy control; CIS: clinically isolated syndrome; MS: multiple sclerosis.

A total of 6017 WMLs were analysed, with 3987 in MS or clinically isolated syndrome (CIS) patients. The mean and interquartile ranges of WMLs per patient found in each condition are represented in Figure 2. Across the analysis, inter-rater reliability for lesion and PRL detection between investigators showed a substantial agreement with a Cohen’s Kappa value of 0.640 and 0.696, respectively. Furthermore, intra-rater reliability had a Cohen’s Kappa value of 0.827.

### Paramagnetic rim lesions

**Figure 3.**
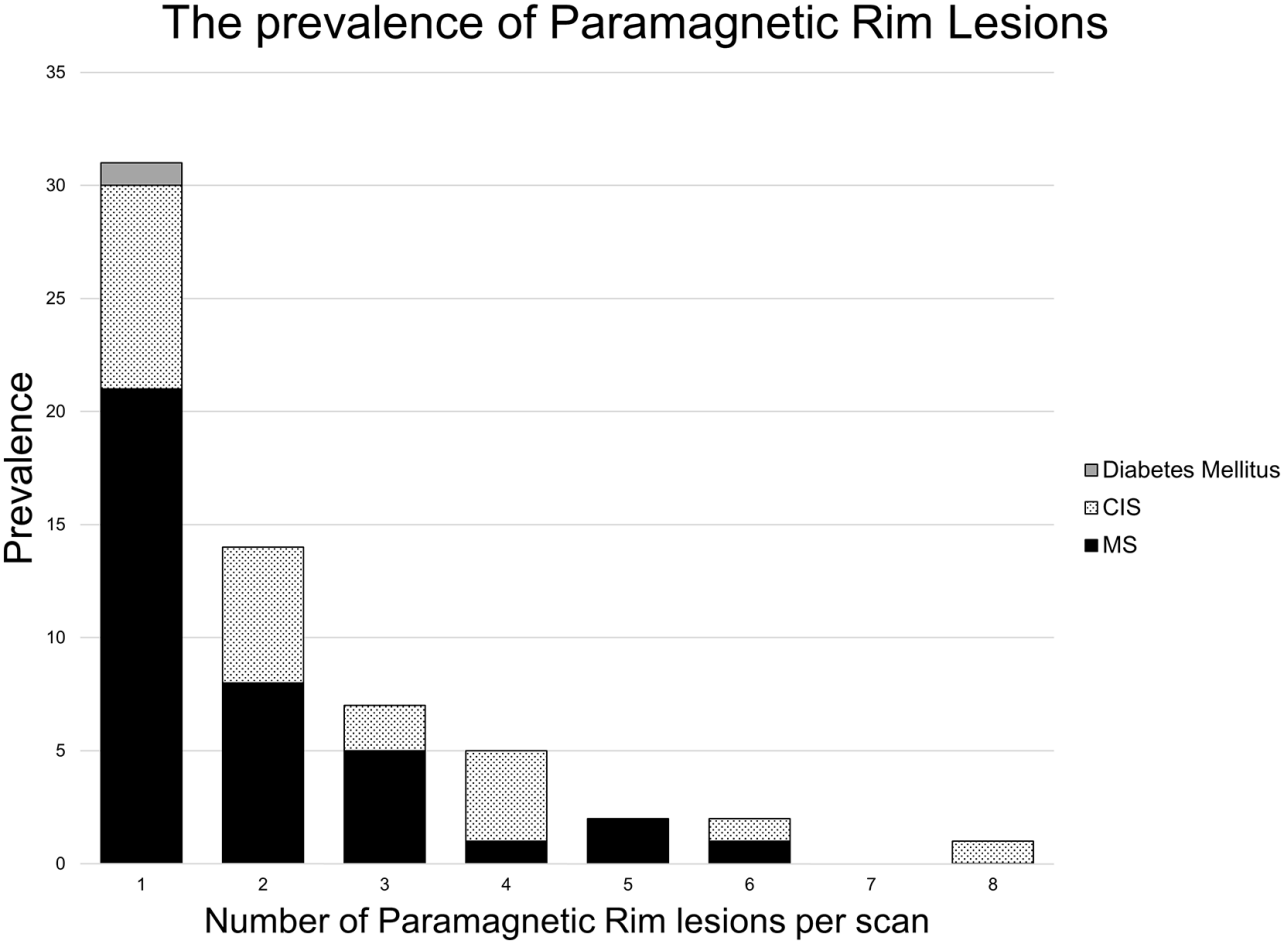
The number of paramagnetic rim lesions per patient for each cohort with ⩾1 paramagnetic rim lesion. CIS: clinically isolated syndrome; MS: multiple sclerosis.

PRLs were detected in 130 lesions across 62 patients. Within the MS cohort, 38 patients (22.9% (CI = 16.7%–30.0%)) had at least one PRL. In the CIS cohort, the proportion of individuals with at least one PRL was 26.1% (CI = 17.3%–36.6%), or 23 patients. Half of PRL positive scans had a single PRL (Figure 3). A single PRL was found in the scan of a diabetic patient, and this was the only PRL detected outside of the MS/CIS cohorts. Although the combined sensitivity of PRL for MS/CIS was 24.0% (CI = 18.9%–29.8%), PRLs had a very high specificity of 99.7% (CI = 98.2%–99.99%) and a PPV (positive predictive value) of 98.39.

All patients with a PRL showing a CVS in the same lesion (*n* = 54) had MS or CIS, giving a specificity of 100% (CI = 98.8%–100.0%) and a PPV of 100. The sensitivity of PRL with a CVS for MS was 20.5% (CI = 12.9%–25.4%) and 22.7% (CI = 14.5%–32.3%) in the CIS patients. In all MS/CIS patients displaying a PRL, 88.5% had a lesion displaying both PRL and CVS. The single PRL detected in the patient with diabetes did not display the CVS.

The identification of ⩾ 1 PRL (optimal cut-off) was associated with high specificity of 99.7%, but low sensitivity of 24.0%, and overall accuracy: area under the curve (AUC) = 0.71, 95% CI = 0.64–0.78. CVS detection alone (optimal cut-off of ⩾ 4 CVS) had specificity of 88.3%, sensitivity of 56.7% and accuracy: AUC = 0.82, 95% CI = 0.79–0.86.

The combination of the two biomarkers (fulfilment of either ⩾ 1 PRL or ⩾ 4 CVS) further improved the specificity (90.6%), and a relative increase in the sensitivity (57.9%). The overall accuracy: AUC = 0.83 (95% CI = 0.79–0.87).

We also performed sequential analysis of the two signs: identification of any PRLs first, and if no PRL was identified followed by assessment of the presence of ⩾4 CVS. The sensitivity of this two-stage analysis was 79.55% (CI = 74.6–83.9) and 70.9% (CI = 64.8–76.4).

Across the cohort, 73.1% of WMLs were found in the deep white matter, 19.4% in the periventricular region, 7.2% were juxtacortical and only 0.3% adjacent to deep grey matter structures. In the MS and CIS cohorts, 70.1% and 66.5% of WMLs were located in the deep white matter, respectively. Yet in the MS cohort, 84.2% of PRLs were identified in the deep white matter. The chi-square test investigating the location of the PRLs and WMLs found PRLs to be more common in the deep white matter (*p* = 0.003).

## Discussion

The PRLs detected in phase-sensitive imaging have potential to aid MS diagnosis. In this article, we expand beyond our original MAGNIMS study of the CVS^14^ to evaluate PRLs using clinically determined 3T MRI protocols. We found the presence of any PRL highly specific for MS/CIS. Furthermore, the combination of PRL with CVS was found only in patients with MS or CIS and not in any other diseases studied with WMLs. Maggi et al.^26^ also reported in their large study low sensitivity and high specificity of PRL in MS. Our study, conducted in different centres, supports their findings. It further adds value as we examined a higher number of patients with MS mimics and ageing controls with brain scans showing WMLs, which more commonly cause diagnostic difficulties for MS clinicians.

In addition to the analysis of the value of PRL and CVS, we performed a sequential analysis (first looking for the presence of PRL, and in the absence of any PRL assessing for ⩾ 4 CVS). Although this did not lead to improvement of sensitivity and specificity of the diagnosis of MS, it may prove popular with MS clinicians as it is time efficient while reviewing MRI scans with WMLs. This sequential analysis of course needs to be tested in a prospective study.

In both Sinnecker et al.^14^ and this analysis, we have recognised that the special distribution of the MS lesions and lesion characteristics may inadvertently un-blind the observer to the diagnosis and influence subsequent lesion characterisation on the same scan. For that reason, we have tried to improve the blinding by parcellating the brain into eight blocks and randomising the order of blocks analysis. In this way, we are certain that the investigators assessed individual lesions without influence of other brain/lesion characteristics.

Although CVS is sensitive to MS, it was found to be less specific than PRL. Both of these imaging biomarkers are acquired on the same MRI sequence and may reduce the need for oligoclonal band testing which many patients find unpleasant. Our study strengthens the evidence for the role of phase-sensitive imaging in the diagnostic pathway of MS.

Our study is pragmatic, with clinical scans acquired by many centres, resulting in variability of scan quality, sequences and operators. The results are therefore representative of the performance of this radiological biomarker in clinical practice. The patient-level prevalence of PRLs is within the range previously described by K.C. Ng Kee Kwong et al.^31^ In this cross-sectional study, we did not aim to report on the natural history of PRLs but found that the percentage of lesions with an iron rim was higher in CIS compared to MS. This corroborates previous longitudinal studies which have suggested that PRLs may eventually dissipate as neuroinflammation is replaced by neurodegenerative pathology.^19,32^

While in our study we examined the role of PRLs in the diagnosis of MS, the debate continues whether most smouldering lesions produce a visible PR. Expanding lesion volume is important since it may be predictive of long-term clinical disability.^18^ Our results suggest that one PRL is enough to help the diagnosis of MS, but counting the number of PRL might be important as a prognostic factor for long-term disability. Studies suggest that some PRLs shrink after 7 years, at which point the iron rim has faded along with the diffuse hyperintensity outside the rim.^13,30^ It would be useful to examine the effect of disease-modifying treatments on PRLs. Unfortunately, data about the multiple disease-modifying treatments used in our cohort is unavailable. Furthermore, the scans available were not taken at the time of diagnosis of MS, thus we have been unable to determine at what point in the disease progression PRLs may be most prevalent.

As this cohort was previously reported, the limitations are similar.^14^ This study relied on the investigators’ clinical diagnosis and we did not independently assess the accuracy of the MS diagnosis or MS by subtype. Some publications suggest that relapsing-remitting and secondary progressive MS have a differing prevalence of PRLs.^33,34^ The parcellated nature of the blocks, although essential for blinding, may also have resulted in lesions not being counted if they were dissected by the border of the blocks. This may account for why not all the MS patients had lesions found on their scans, although we also excluded lesions smaller than 3 mm in their longest axis. The parcellation method used to truly blind the investigators might have resulted in the moderate reproducibility we report. We suspect that in clinical practice, clinicians will be influenced also by other MRI diagnostic features of the WM lesions. Using automated techniques may prove to be beneficial in improving the accuracy of PRL detection by eliminating human errors. Once again only prospective studies can assess the true diagnostic value of a test.

## Conclusion

Paramagnetic rims are a potential imaging biomarker, with high diagnostic specificity for MS. They have a clinical role to play in decreasing the diagnostic uncertainty in MS. In this large study, a quarter of MS/CIS patients had at least one PRL. Furthermore, 3T phase-sensitive MRI is widely available and has already been proven to reliably identify the CVS. The combination of these radiological markers detected with the same MRI sequence shows great promise and requires further prospective evaluation, perhaps with added improvements to sequence optimisation.

## Supplemental Material

sj-docx-1-msj-10.1177_13524585221118677 – Supplemental material for Paramagnetic rims are a promising diagnostic imaging biomarker in multiple sclerosis Click here for additional data file. Supplemental material, sj-docx-1-msj-10.1177_13524585221118677 for Paramagnetic rims are a promising diagnostic imaging biomarker in multiple sclerosis by Isobel Meaton, Amjad Altokhis, Christopher Martin Allen, Margareta A Clarke, Tim Sinnecker, Dominik Meier, Christian Enzinger, Massimiliano Calabrese, Nicola De Stefano, Alain Pitiot, Antonio Giorgio, Menno M Schoonheim, Friedemann Paul, Mikolaj A Pawlak, Reinhold Schmidt, Cristina Granziera, Ludwig Kappos, Xavier Montalban, Àlex Rovira, Jens Wuerfel and Nikos Evangelou in Multiple Sclerosis Journal
