# Supplementary material for: Paramagnetic rims are a promising diagnostic imaging biomarker in multiple sclerosis
Source: Mult Scler. 2022 Aug 26;28(14):2212–20. doi: 10.1177/13524585221118677 (PMC9679799; doi:10.1177/13524585221118677)
Supplement: sj-docx-1-msj-10.1177_13524585221118677 – Supplemental material for Paramagnetic rims are a promising diagnostic imaging biomarker in multiple sclerosis [file sj-docx-1-msj-10.1177_13524585221118677.docx]

**Supplementary ROC - Supplemental material for “Paramagnetic rims are a promising diagnostic imaging biomarker in multiple sclerosis”.**

**Supplemental material, Supplementary ROC, for “Paramagnetic rims are a promising diagnostic imaging biomarker in multiple sclerosis” by Isobel Meaton, Amjad Altokhis, Christopher Martin Allen, Margareta A Clarke, Tim Sinnecker, Dominik Meier, Christian Enzinger, Massimiliano Calabrese, Nicola De Stefano, Alain Pitiot, Antonio Giorgio, Menno M Schoonheim, Friedemann Paul, Mikolaj A Pawlak, Reinhold Schmidt, Cristina Granziera, Ludwig Kappos, Xavier Montalban, Àlex Rovira, Jens Wuerfel, and Nikos Evangelou on behalf of the MAGNIMS study group, in Multiple Sclerosis Journal.**

Receiver operating characteristic curves (ROC) for prediction the diagnosis of MS vs MS-mimics based on Central Vein Sign (CVS) and Paramagnetic Rim Lesion (PRL).

Figure 1. Illustrates the ROC curve analysis for the Central vein sign and Figure 2. Illustrates the CVS and PRL combined which has no significant difference.


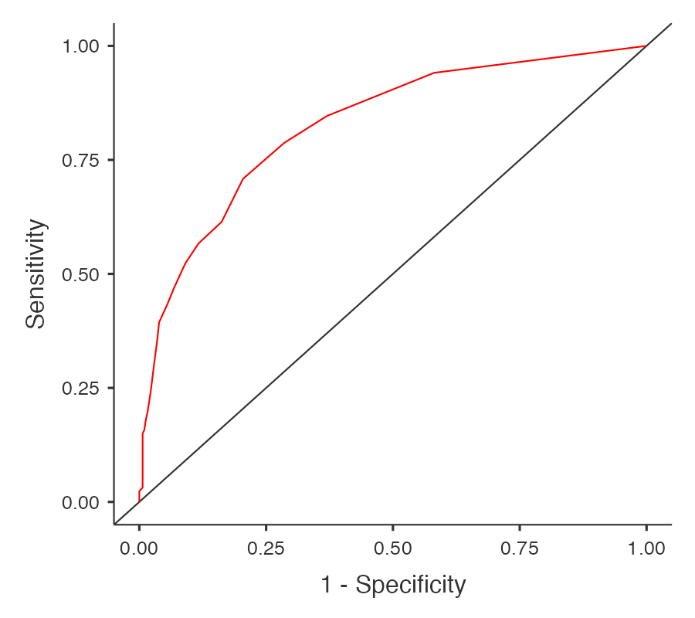


Figure 1. ROC for the dependent variable of diagnosis (MS vs MS-mimics) against the independent variable of Central Vein Sign


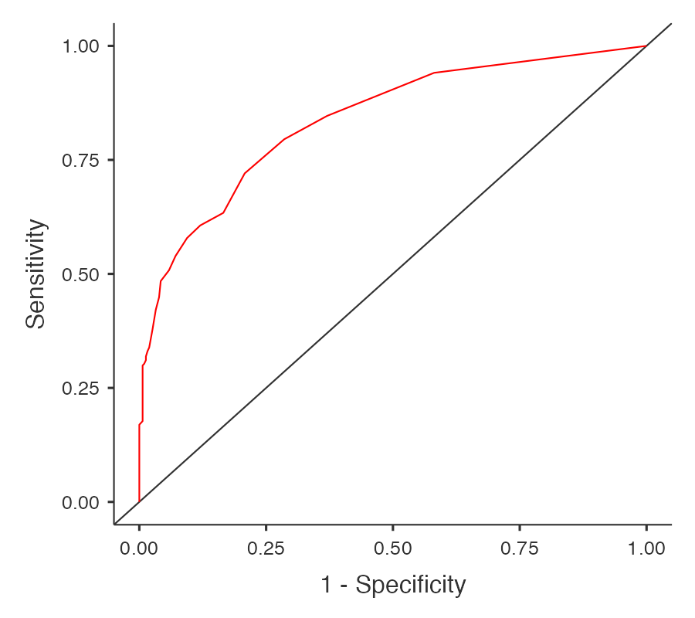


Figure 2. ROC for the dependent diagnosis (MS vs MS-mimics) against the independent variable of Paramagnetic Rim Lesions or Central Vein Sign
